# Addition of Laponite to gelatin methacryloyl bioinks improves the rheological properties and printability to create mechanically tailorable cell culture matrices

**DOI:** 10.1063/5.0166206

**Published:** 2024-01-08

**Authors:** Jordan W. Davern, Luke Hipwood, Laura J. Bray, Christoph Meinert, Travis J. Klein

**Affiliations:** 1 Centre for Biomedical Technologies, Queensland University of Technology (QUT), Brisbane, QLD, Australia; 2 School of Mechanical, Medical and Process Engineering, Queensland University of Technology (QUT), Brisbane, QLD 4059, Australia; 3 ARC Training Centre for Cell and Tissue Engineering Technologies, Queensland University of Technology (QUT), Brisbane, QLD 4059, Australia; 4 Gelomics Pty Ltd, Brisbane, QLD 4059, Australia; 5 Faculty of Health, School of Biomedical Sciences, Queensland University of Technology (QUT), Brisbane, QLD 4059, Australia

## Abstract

Extrusion-based bioprinting has gained widespread popularity in biofabrication due to its ability to assemble cells and biomaterials in precise patterns and form tissue-like constructs. To achieve this, bioinks must have rheological properties suitable for printing while maintaining cytocompatibility. However, many commonly used biomaterials do not meet the rheological requirements and therefore require modification for bioprinting applications. This study demonstrates the incorporation of Laponite-RD (LPN) into gelatin methacryloyl (GelMA) to produce highly customizable bioinks with desired rheological and mechanical properties for extrusion-based bioprinting. Bioink formulations were based on GelMA (5%–15% w/v) and LPN (0%–4% w/v), and a comprehensive rheological design was applied to evaluate key rheological properties necessary for extrusion-based bioprinting. The results showed that GelMA bioinks with LPN (1%–4% w/v) exhibited pronounced shear thinning and viscoelastic behavior, as well as improved thermal stability. Furthermore, a concentration window of 1%–2% (w/v) LPN to 5%–15% GelMA demonstrated enhanced rheological properties and printability required for extrusion-based bioprinting. Construct mechanical properties were highly tunable by varying polymer concentration and photocrosslinking parameters, with Young's moduli ranging from ∼0.2 to 75 kPa. Interestingly, at higher Laponite concentrations, GelMA cross-linking was inhibited, resulting in softer hydrogels. High viability of MCF-7 breast cancer cells was maintained in both free-swelling droplets and printed hydrogels, and metabolically active spheroids formed over 7 days of culture in all conditions. In summary, the addition of 1%–2% (w/v) LPN to gelatin-based bioinks significantly enhanced rheological properties and retained cell viability and proliferation, suggesting its suitability for extrusion-based bioprinting.

## I. INTRODUCTION

Three-dimensional (3D) bioprinting combines cells, molecules, and biomaterials to precisely pattern and assemble tissue-like structures.^1,2^ 3D bioprinting has been utilized in the fields of tissue engineering, regenerative medicine, and disease modeling to recapitulate key characteristics of various native tissues.^3^ A popular method of bioprinting is extrusion-based, where a bioink is forced through a nozzle to create controlled 3D constructs in a layer-by-layer fashion.^1,3^ Over the last decade, many biomaterials [e.g., gelatin methacryloyl (GelMA), polyethylene glycol (PEG), alginate, or collagen] have been developed into bioink formulations for bioprinting. The major challenges with adapting these materials for bioprinting lie in balancing the mechanical, rheological, chemical, and biological properties such the bioinks are both printable and biocompatible (i.e., within the “biofabrication window”).^4^ Most traditional biomaterials utilized for 3D cell culture lack the required rheological properties for extrusion-based printing and must be modified to meet the bioprinting demands.^5–7^ For example, GelMA undergoes thermally induced gelation at temperatures below 30 °C, dependent upon polymer concentration, resulting in changes in viscosity and inconsistent print fidelity.^8–10^ To mitigate this limitation, temperature-controlled print beds and syringe barrels can be used. Alternatively, GelMA formulations can be modified by incorporating additives to improve the temperature-dependent behavior and enhance printability.^1–4,9,11–13^ Yet, limited bioinks are readily available for the development of soft matrices (<20 kPa) using extrusion-based bioprinting.^3,5,6,11,12,14–21^

Given the central role of rheology in the success or failure of a bioink, thorough rheological analysis is an efficient pathway to determine the suitability of prospective bioinks for extrusion-based printing.^11^ Several key rheological parameters should be considered during ink development, including viscosity, shear thinning, thixotropy, and temperature range required for stable bioprinting. Viscosity plays an important role in cell sedimentation, aggregation, and shear stresses during and after extrusion.^3,16^ Shear thinning is the non-Newtonian decrease in material viscosity with increasing shear rate resulting from the reorganization of polymer chains during the extrusion through the printing nozzle.^14,15^ Shear thinning bioinks can be deposited under lower extrusion pressures and lower shear stresses which may otherwise impact cell viability.^6,16,17^ Thixotropy refers to time-dependent shear thinning and can be associated with the viscoelastic properties of a material.^3,11,12,18^ In extrusion-based bioprinting, inks deposited onto desired surfaces require rapid recovery (non-thixotropic) and enhanced viscoelasticity to retain structural integrity with increasing layers being deposited. Together, these parameters influence yield stress, defined as the pressure required to overcome and initiate flow to dispense material.^19^ Another key parameter to consider during ink development is the temperature window required and is dependent upon biomaterial choice and printing outside of the window may result in reduced rheological properties and poor shape fidelity. Implementing strategies that facilitate shear thinning and non-thixotropic recovery enables increased printability whilst maintaining cellular viability.^5,15,20,21^

To advance 3D cell culture biomaterials into bioinks, various approaches have been explored. These include incorporating secondary polymers to enhance the main polymer formulations, utilizing supramolecular hydrogels, and forming nanocomposites.^14^ Substances such as alginate, kappa carrageenan, nanocellulose, and nanoclays have proven effective as rheological modifiers to improve the print fidelity of base materials such as PEGDA and GelMA, however they have their own disadvantages.^5,18,22–26^ For example, alginate and kappa carrageen lack cell adhesion ligands, and have poor cross-linking kinetics.^27^ While composites of GelMA and kappa carrageenan have been utilized to improved shape fidelity, the increased viscosity, shear stress and mechanical strength limit its tissue specificity.^28,29^ These modifiers improve shear thinning behavior and enhance print fidelity compared to non-modified bioink formulations, while maintaining similar cell viability and function.^8,26,30,31^ Temperature sensitivity and fluctuations still pose a limitation with current rheological additives with a focus shifted toward the introduction of temperature inert particles for enhanced stability and incorporation with currently available biomaterials.

Nanoparticles show promise as a material class capable of generating colloidal-like suspensions within aqueous hydrogel formulations to enhance the rheological properties of bioinks, enabling the generation of advanced bioinks capable of both high fidelity and cytocompatibility.^30,32–34^ Laponite (LPN), a smectite nanomaterial commonly used within the cosmetic industry, has been utilized as a rheological additive for osteogenic and vascular bioinks.^28,30,32,35^ LPN are disk-shaped particles with a diameter of approximately 25 nm and thickness of 1 nm, and heterogeneity of its charge with negative face charges and positive rim charges.^32,36,37^ Once dispersed in aqueous solution, LPN nanoparticles self-organize via face-edge aggregation to form reversible thixotropic gels.^25,32,35^ LPN has been shown to enhance mechanical, and physical properties and contribute to extracellular matrix (ECM) remodeling.^25,35^ Previous studies have demonstrated LPN's capacity for drug and growth factor delivery, providing a customizable platform that can be tailored to each specific application, such as prolonged release of recombinant human bone morphogenetic protein 2 (rhBMP2) or transforming growth factor-β3 (TGF-β3), or tetracycline delivery for periodontal disease.^38,39^

We hypothesized that adding LPN to GelMA would alter shear-thinning, temperature sensitivity and viscoelastic characteristics required for extrusion-based printing. Here, we demonstrate the incorporation of 1%–4% (w/v) LPN to 5%–15% (w/v) GelMA to enhance its rheological properties and printability in extrusion-based bioprinting. The concentrations of 0%–4% (w/v) LPN were selected based on previous studies, whereas 5%–15% (w/v) GelMA was selected based on the versatile tissue stiffness (elastic modulus) that can be matched, such as endothelial (∼1 kPa) to musculoskeletal tissues (∼150 kPa) for the development of multi-tissue specific bioinks.^25,29,32,35,40,41^ In previous studies, the use of 0%–6% (w/v) LPN in gelatin-based bioinks has been explored, primarily focusing on shear-thinning and temperature behavior.^25,35,42–45^ In contrast, the current study aims to narrow down this concentration range. It employs a comprehensive rheological approach to investigate the ideal LPN concentration for incorporation into gelatin-based bioinks, considering various rheological properties in detail. This study focuses on a comprehensive rheological analysis to investigate the addition of LPN to GelMA and its influence on shear-thinning, temperature sensitivity, viscoelasticity, mechanical properties, and printability. Finally, MCF-7 human breast cancer cell viability and spheroid formation is assessed to determine GelMA-LPN's suitability for *in vitro* cancer models.

## II. RESULTS

**FIG. 1.**
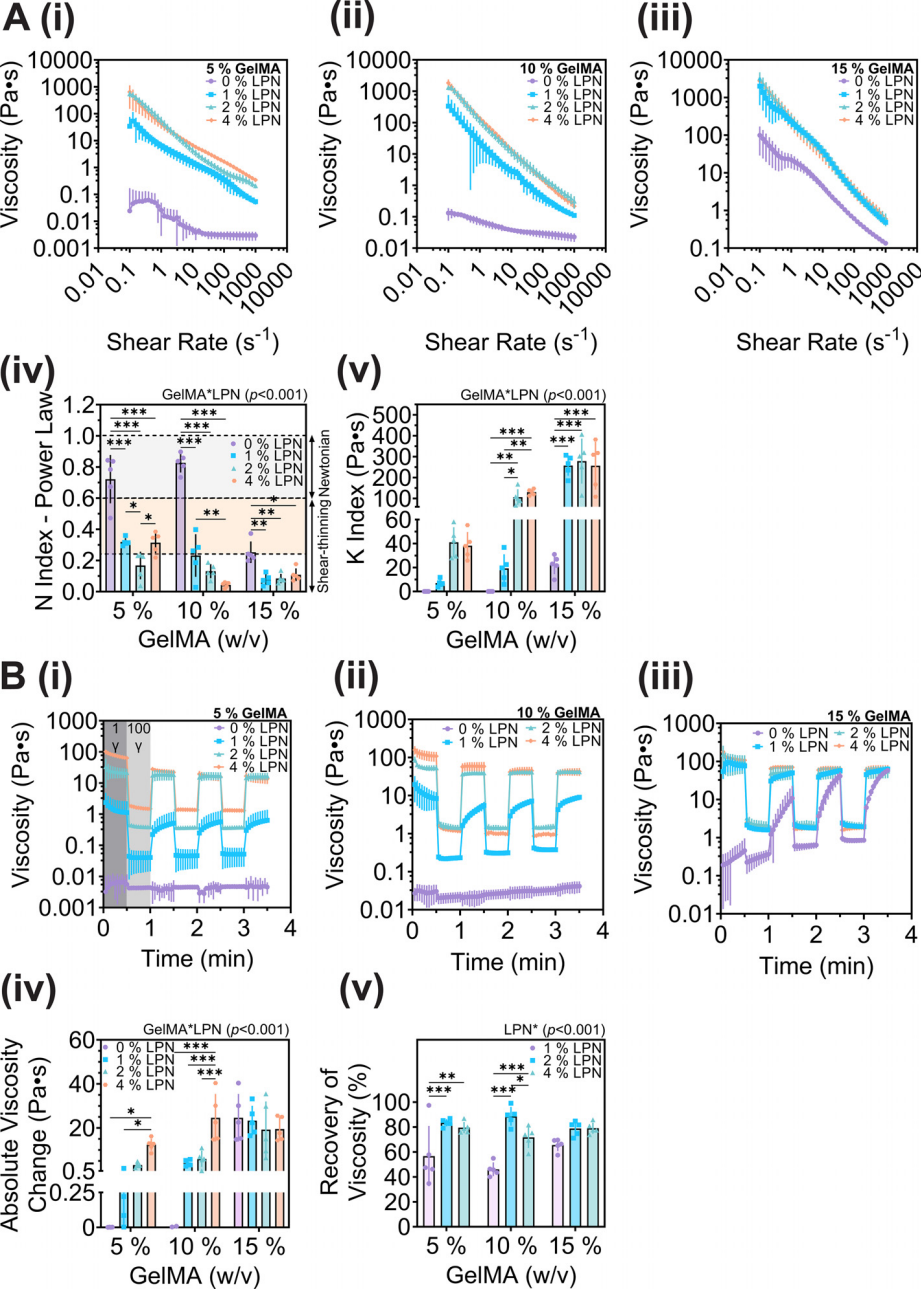
Viscosity profiles and 3-interval thixotropy test of GelMA-LPN bioinks. (a) Viscosity shear rate (0.1–1000 s^−1^) ramps of 5%–15% GelMA with the addition of 0%–4% (w/v) Laponite (LPN) (i)–(iii) were performed to assess viscosity and shear shear-thinning behavior using power law regression model *n* index (Newtonian = 1, shear-thinning < 1, and shear thickening > 1), and *K* index (flow consistency index) (iv) and (v). (b) A 3-interval viscosity test during low shear (1 s^−1^) and high shear rate (100 s^−1^) cycles of 30 s intervals of 5%–15% GelMA with 0%–4% (w/v) Laponite (i)–(iii) investigated the viscoelasticity and shape recoverability, (iv) average recovery of viscosity refers to the change (%) of viscosity from initial pre-shear phase to post high shear recovery. (v) The absolute viscosity refers to the initial recovery viscosity measurement change once high shear rate has been applied. Mean values with error bands indicating standard deviation (SD), n = 5 for all groups. Statistical differences were performed using two-way ANOVA with Tukey post-hoc test (^*^P < 0.05; ^**^P < 0.01; ^***^P < 0.001).

To determine LPN's influence on the rheological properties of GelMA bioinks, a series of rheological tests were undertaken. Bioinks demonstrated increased viscosity and shear-thinning behavior across all GelMA concentrations (5%–15% w/v) in the presence of LPN (1%–4%) compared to LPN-free controls [Figs. 1(a-i)–1(a-iii)]. A power law model was utilized to further evaluate LPN's influence using the *n* index and *K* (flow consistency index) [Eq. (1)]. Measures of *n* index characterize flow behavior (Newtonian fluids = 1, shear-thinning < 1, and shear-thickening > 1) and *K* index refers to the viscosity gradient of a fluid to shear rate. The addition of 2% and 4% LPN across all tested GelMA concentrations (5, 10, or 15%, w/v), respectively, significantly increased shear-thinning capacity with an *n* index of <0.25 and increased *K* index ranging from 40 to 300 Pa s [Figs. 1(a-iv) and 1(a-v)], suggesting LPN enhances GelMA's initial viscosity whereupon shear is applied resulting in shear thinning.

Thixotropic tests were performed to simulate the extrusion process and determine ink shape recoverability post-extrusion using low (1 s^−1^) and high (100 s^−1^) shear rate cycles. Thixotropic responses (time-dependent shear thinning) were observed in 5%–15% GelMA containing ≤1% LPN, while groups containing ≥2% (w/v) LPN exhibited non-thixotropic behavior [Figs. 1(b-i)–1(b-iii)], suggesting the higher LPN concentration bioinks would better retain their shape post-printing. The absolute viscosity, which is referred to as the initial change of viscosity during recovery phase (1 s^−1^), was calculated to evaluate the material's viscoelastic and shear thinning properties. The GelMA controls (5%–10% w/v) elicited no significant change of viscosity during initial recovery phase, further demonstrating viscoelastic liquid characteristics and poor recoverability. 15% (w/v) GelMA exhibited a similar thixotropic trend, however, viscosity increased because of the high polymer concentration resulting in sol-gel formation from prolonged shear [Figs. 1(b-iii)–1(b-v)]. The absolute viscosity increased 2.89–24.61 Pa s in 5%–15% GelMA groups containing ≥2% (w/v) and all groups had ≥75% recovery during low shear rate phases [Figs. 1(b-iv)–1(b-v)].

**FIG. 2.**
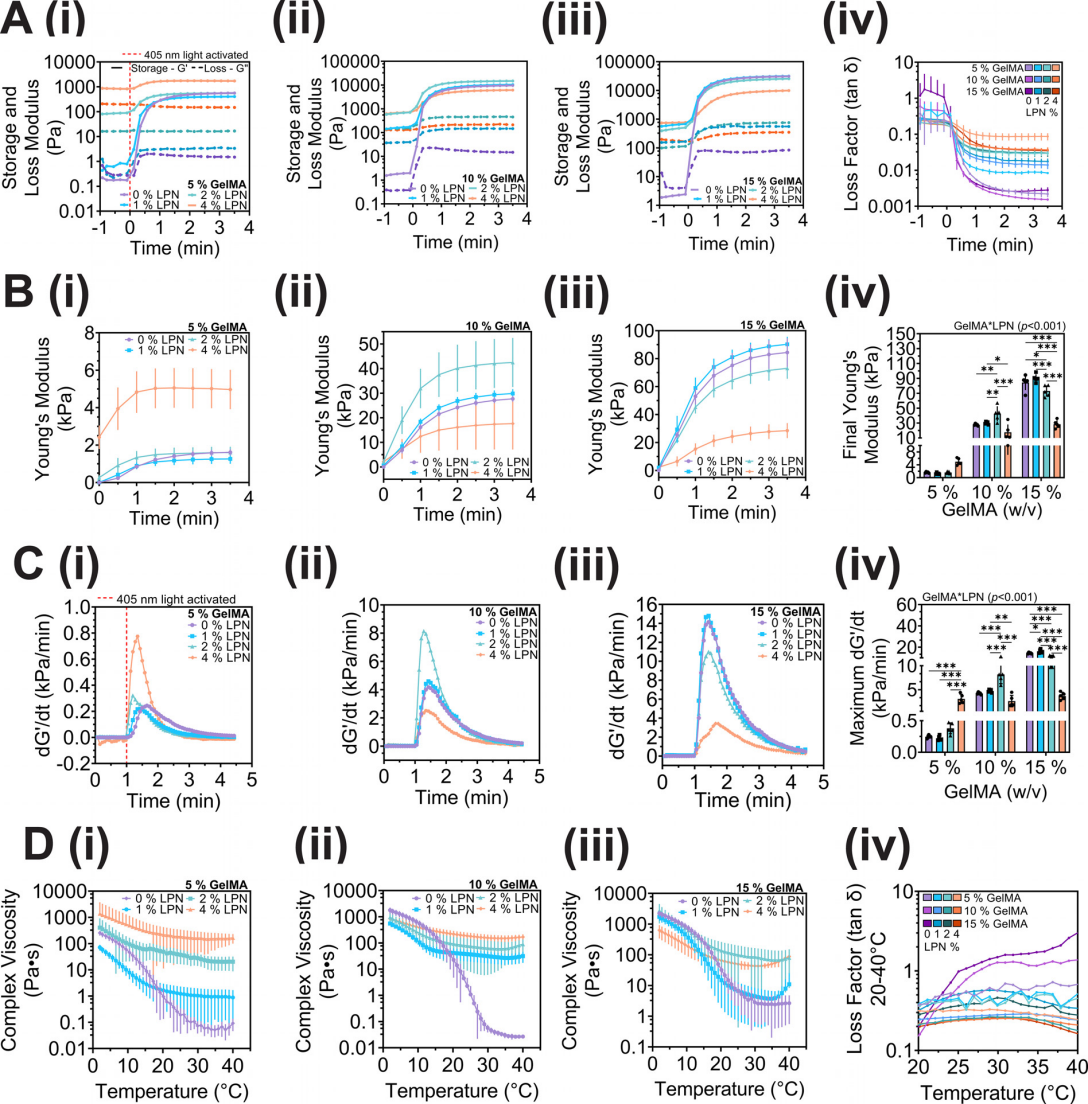
Photorheology and temperature sweep of bioinks containing 5%–15% GelMA and 0%–4% LPN. (a) Oscillatory-time sweeps of 5%–15% GelMA with 0%–4% (w/v) Laponite (LPN) measuring storage modulus (G′) and loss modulus (G″) during cross-linking of precursor solutions using visible light source (405 nm) at 1 min (red dashed lined) (i)–(iii) and the calculated loss factor (tan *δ*) of all GelMA-LPN concentrations (iv). (b) Young's moduli of 5%–15% GelMA with 0%–4% (w/v) LPN. Time (*t*) at 0 min indicates commencement of visible light activation (i)–(iii). Final Young's modulus corresponds to the last recorded measurement (t = 3.5 min) (iv). (c) Crosslinking propagation (Δ storage modulus–G′/time—minutes) of 5%–15% GelMA with 0%–4% (w/v) LPN (i)–(iii) hydrogels and maximum cross-linking reaction (iv). (d) Temperature sweep of 5%–15% GelMA with 0%–4% (w/v) LPN (i)–(iii) within a 40–2 °C temperature range at a rate of 2 °C/min to assess LPN's influence on GelMA's printing window and viscoelastic behavior using loss factor (tan *δ*) within 20–40 °C (iv). Mean values are represented with or without error bands indicating standard deviation (SD) and sample size (n) = 5 for all groups. Statistical differences were performed using two-way ANOVA with Tukey post-hoc test (^*^P < 0.05; ^**^P < 0.01; ^***^P < 0.001).

To assess the cross-linking kinetics of GelMA-based bioinks with and without LPN, oscillatory rheological tests were performed with 405 nm photocrosslinking of bioinks initiated after 1 min. The storage and loss moduli increased across all groups upon light activation, greater change was observed in 5%–15% (w/v) GelMA controls further highlighting the transition from viscoelastic-liquid to solid, compared to 5%–15% GelMA with 1%–4% (w/v) LPN which already exhibited viscoelastic-solid behavior (storage modulus > loss modulus) prior to cross-linking [Figs. 2(a-i)–2(a-iv)]. The cross-linking kinetics, derived from the storage modulus and rate of propagation,^47^ were investigated to determine the influence of LPN on the network formation. Bioinks containing 5%–10% GelMA with the addition of 1 or 2% LPN, respectively, elicited an increase in mechanical properties compared to 5%–10% (w/v) GelMA controls [Figs. 2(b-i)–2(b-iii)]. The final Young's moduli after 3.5 min of photocrosslinking of LPN-free controls were 1.61 ± 0.16 and 27.7 ± 1.17 kPa for 5% and 10% GelMA, respectively, and groups containing 2% LPN measuring 1.59 ± 0.31 (5% GelMA) and 42.5 ± 9.9 (10% GelMA) [Fig. 2(b-iv)]. The incorporation of 4% LPN in 10%–15% (w/v) GelMA, respectively, decreased mechanical properties and rate of storage modulus propagation (dG′/dt) compared to lower concentrations of 0%–2% (w/v) LPN [Figs. 2(b-i)–2(b-iv) and 2(c-i)–2(c-iv)].

The addition of 4% LPN increased cross-linking propagation within 5% GelMA and led to increased Young's modulus compared to 5% GelMA with 0%–2% LPN (w/v) [Figs. 2(c-i) and 2(b-iv)]. Conversely, the addition of 4% LPN to high polymer concentrations of 10%–15% (w/v) GelMA significantly reduced rate of propagation [Figs. 2(c-ii)–2(c-iv)] and Young's modulus [Fig. 2(b-iv)]. The addition of 2%–4% LPN across all GelMA concentrations improved stability of viscosity within the desired print temperature range (22.5–26 °C) [Figs. 2(d-i)–2(d-iv)]. The complex viscosity and tan *δ* of LPN-containing groups resulted in limited fluctuation (tan *δ* 0.56–0.21) within the temperature range of 22–37 °C reflecting its viscoelastic solid behavior and further enhancing GelMA for extrusion-based printing [Figs. 2(d-i)–2(d-iv)].

**FIG. 3.**
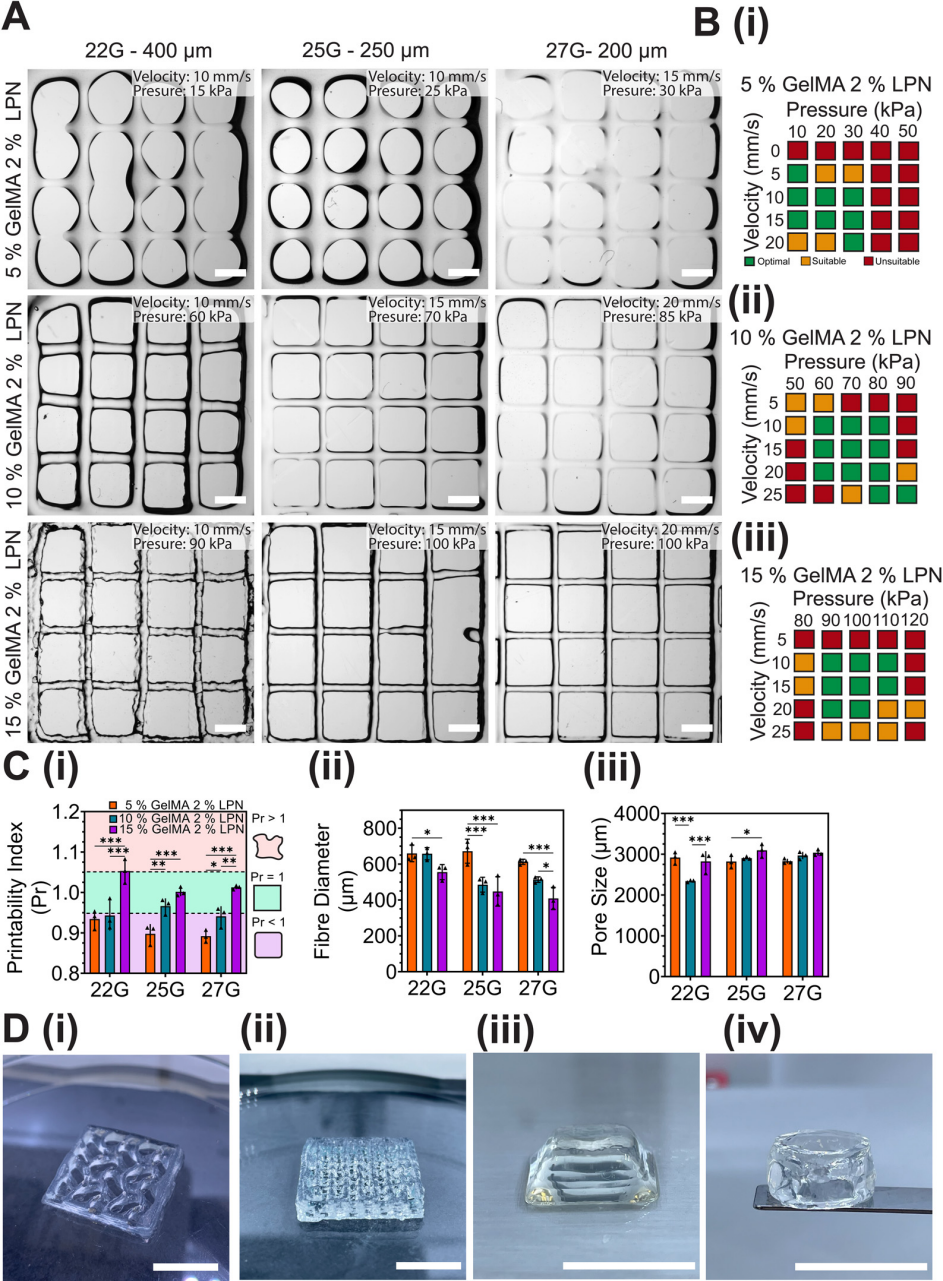
Print characterization of selected bioinks containing 5%–15% GelMA and 2% (w/v) LPN. (a) Lattice-like structures (12 × 12 mm^2^) printed using conical needle sizes (22 G—400 *μ*m, 25 G—250 *μ*m, 27 G—200 *μ*m) of GelMA 5%–15% with 2% (w/v) LPN, scale bar = 2 mm. (b) Printing speed and extrusion pressure range of 5%–15% GelMA and 2% (w/v) LPN using 22–25 G conical nozzles. Colored boxes indicate level of printability using print speed and pressure ranging from unsuitable (red), suitable (amber), and optimal (green), all represented constructs were printed within optimal (green) settings. (c) Quantitative analysis using Image J of GelMA 5%–15% with 2% (w/v) LPN using 22–27 G nozzles showing: (i) Pr, with green shading indicates ideal Pr values = 1 ± 0.05; (ii) measured fiber diameter; and (iii) pore size. (d) Printed varied geometrical constructs using 10% GelMA and 2% (w/v) LPN (i) 5-layered 15 × 15 mm^2^ construct (ii) 10-layered 15 × 15 mm^2^ lattice constructs and (iii) and (iv) various 3D-printed shapes left-right, trapezium and cylinder. The mean values are represented with error bands indicating standard deviation (SD) and sample size (n) = 3–5 for all groups. Statistical analyses were performed using two-way ANOVA with Tukey post-hoc test (^*^P < 0.05; ^**^P < 0.01; ^***^P < 0.001).

The rheological investigation demonstrated that inclusion of LPN resulted in the enhancement of crucial rheological properties of GelMA that are necessary for extrusion-based bioprinting. These properties included shear-thinning behavior (*n* index ≤ 0.16), shape recovery (≥82.5%), mechanical properties (0.26–73.10 kPa), and reduced temperature-dependent fluctuations of viscosity (tan *δ*: 0.17–0.55). The study determined that 2% (w/v) LPN showed the most significant improvement in these properties. Following selection of 2% LPN as the best additive concentration for 5%–15% (w/v) GelMA, studies were commenced using different conical nozzles (22 G—400 *μ*m to 27 G—200 *μ*m) to assess their influence on print fidelity of simple lattice structures. The study revealed that the size of the nozzle had an impact on the print fidelity. Specifically, an overall decrease in fiber diameter and pore size was observed as the concentration of GelMA increased within nozzle sizes 25–27 G, in contrast to 22 G which resulted in the printing of inconsistent structures [Fig. 3(a)].

A printability index (Pr) value = 1 corresponds to a defined square shape and ±0.05 is an accepted margin of printability,^48^ while Pr > 1.05 indicates over-gelation and irregular structures, Pr < 0.95 signifies a lack of gelation and overly circular pores.^18^ When using 25–27 G nozzles for extrusion of 10%–15% GelMA with 2% (w/v) LPN, Pr was close to ideal (10% GelMA: Pr ≈ 0.94, 15% GelMA: Pr ≈ 1.01). Using larger nozzle size (22 G) or lower GelMA concentration (5% w/v) led to under-gelation (Pr < 1) of printed constructs [Fig. 3(c-i)]. Decrease of nozzle diameter (22 to 27 G) resulted in an increase in pore size and decrease in fiber diameter across 10%–15% (w/v) GelMA [Figs. 3(c-ii) and 3(c-iii)]. Higher polymer (GelMA) concentrations required increased pressure to extrude, while the print velocity (mm/s) had limited effect on bioink filament extrusion. 10% GelMA with 2% (w/v) LPN had a wider optimal print window when pressure and print velocity values were adjusted compared to 5% and 15% GelMA [Figs. 3(b-i)–3(b-iii)]. The 25–27 G nozzle sizes resulted in consistent and highly accurate printed constructs using 10% GelMA and 2% (w/v) LPN, with the ability to print a variety of geometries and heights [Fig. 3(d)].

**FIG. 4.**
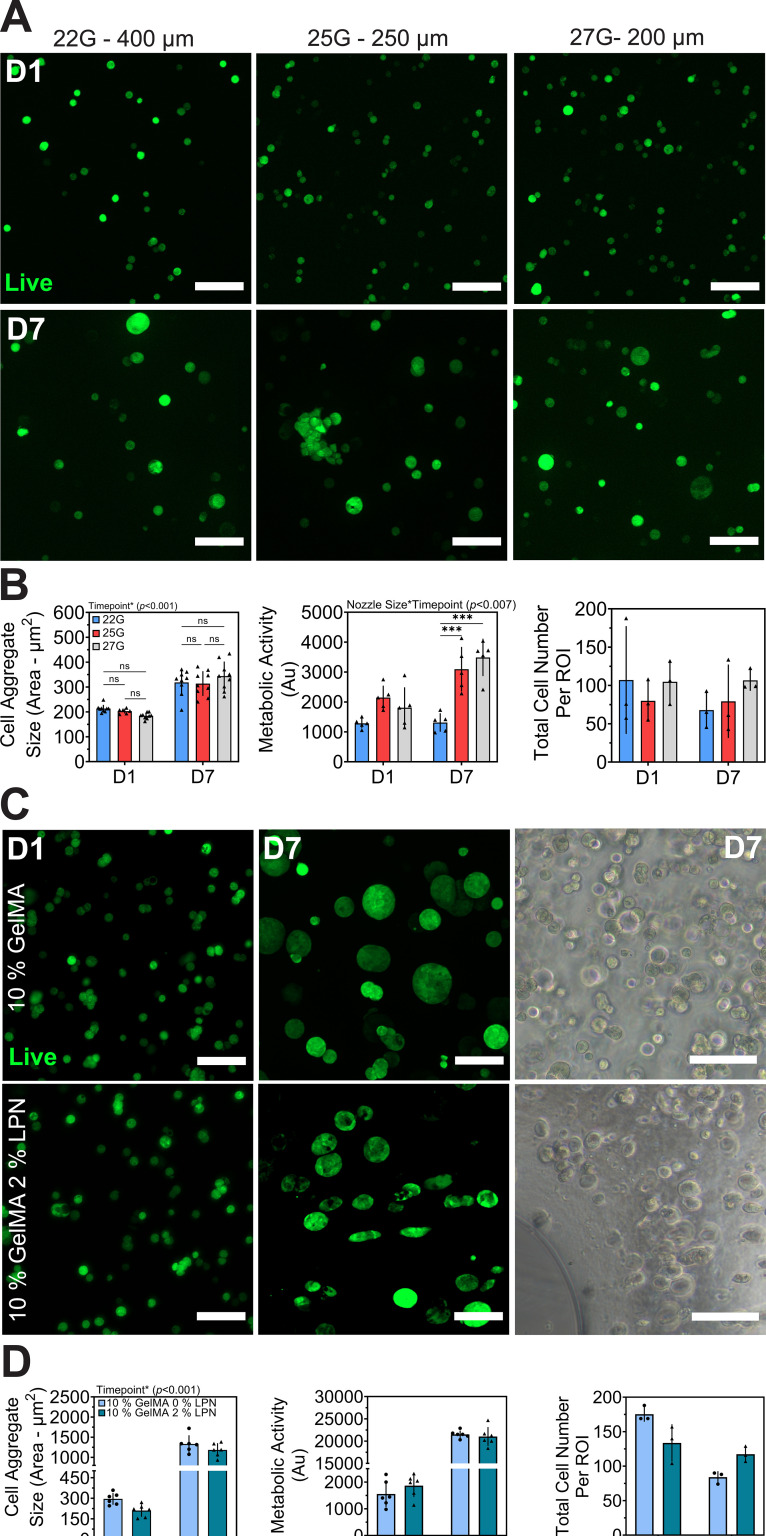
MCF-7 breast cancer cell viability in 3D constructs of 10% GelMA with 0%–2% (w/v) LPN. (a) Maximum intensity projections assessing viability over 7 days in 10% GelMA with 2% (w/v) LPN printed using different nozzle sizes (22 G—400 *μ*m, 25 G—250 *μ*m, 27 G—200 *μ*m) and photocrosslinked post printing using 405 nm light source at light intensity of 14 mW/cm^2^ for 1 min (11.07 kPa). (b) Cell aggregate size and PrestoBlue metabolic activity of constructs printed with 22–27 G nozzles over 7 days. (c) Maximum intensity projection assessing viability and brightfield microscopy of MCF-7 cells encapsulated in 10% GelMA control and 10% GelMA with 2% (w/v) LPN over 7 days using 27 G—200 *μ*m nozzle and crosslinked to match stiffness of 1 kPa (10% GelMA for 1 min and 10% GelMA 2% w/v LPN for 30 s). (d) Cell aggregate size, PrestoBlue metabolic activity and total cell number (averaged from three ROIs per sample) over 7 days. Mean ± SD (n = 4–8), scale bar = 200 *μ*m.

An initial cytocompatibility study was carried out over 7 days to evaluate the cytocompatibility of 5% GelMA and 2% (w/v) LPN in 20 *μ*l free-swelling droplets to determine LPN's impact on breast cancer cell line MCF-7's spheroid formation and metabolic activity (Fig. S2). The addition of LPN increased the formation of metabolically active spheroid and aggregates compared to 5% (w/v) GelMA control (Fig. S2). The addition of LPN affected fluorescent imaging of live and fixed samples, resulting in increased background fluorescence within GelMA-LPN groups and inconsistent acquisition of images using lasers 365, 561, and 633 nm (Fig. S3). As a result, FDA alone was utilized to visualized live cells. Next, a secondary study was conducted over 7 days to evaluate the cytocompatibility of printable ink concentration [10% GelMA with 2% (w/v) LPN] and the impact of nozzle size (22, 25, and 27 G) on MCF-7 cells with 1 min cross-linking time (*E* = 11.07 kPa). Cell morphology changed over time, with individual cells forming rounded spheroids that increased in size and metabolic activity over time [Figs. 4(a) and 4(b)]. Despite variations in nozzle size, there were no significant differences in spheroid/and cell aggregate size whereas significant differences in metabolic activity was observed on day 7 [Fig. 4(b)]. A viability assessment was performed, comparing lattice constructs printed using a 27 G nozzle of 10% GelMA with 2% LPN and free-swelling droplets of 10% (w/v) GelMA with matching stiffness (*E* = 1 kPa) [Figs. 4(c) and 4(d)]. The initial cell size and metabolic activity between the GelMA droplet control and the printed construct were similar. At day 7, both groups demonstrated cell aggregate/spheroid formation resulting in reduce total cell number and increased metabolic activity compared to day 1, suggesting proliferation of MCF-7 cells [Figs. 4(c) and 4(d)].

## III. DISCUSSION

3D bioprinting technology allows for the precise arrangement of cells and biomaterials to fabricate highly organized structures that mimic *in vivo* conditions.^1–3^ However, the progress of extrusion-based bioprinting is hindered by the requirement for specific bioink properties that permit the creation of tissue-like constructs with physiological complexity.^49^ To overcome these limitations, it is imperative to enhance the current bioink standards and expand the biofabrication window through the development of advanced bioinks that exhibit both high resolution and cellular compatibility.^1,3,5,14,15^ Rheological analysis provides a prescreening methodology for evaluating the potential of bioinks, determining if they possess the critical properties required for extrusion-based bioprinting. In the current study, we conducted a rheology-focused investigation to assess the influence of LPN on GelMA bioink formulations to reduce temperature sensitivity and improve printability within the biofabrication window.

LPN is smectite mineral nanoclay which is commonly used within the cosmetic industry due to its ability to enhance the stability and viscosity of emulsions. In addition to its cosmetic applications, LPN has also been utilized as a rheological additive to improve the properties of bioinks for 3D bioprinting.^30,35^ Incorporation of LPN has been observed in various materials, including gelatin, methylcellulose, and most notably GelMA, at concentrations ranging from 0.5 to 8% (w/v).^25,30,35,44,45^ Addition of LPN at concentrations greater than 1% (w/v) to gelatin or GelMA resulted in strong electrostatic interaction between LPN's positively charged rim and negatively charged functional groups (e.g., carboxylic acid), and highly shear-thinning bio-printable compositions.^42,50–52^ Addition of LPN to GelMA improved its shear-thinning capability (n index ≤ 0.25, highly shear-thinning) [Fig. 1(a)], viscoelasticity and shape recoverability [∼80%, Fig. 1(b)], mechanical properties [Fig. 2(b)], and thermal stability [Fig. 2(d)], coinciding with previous studies.^30,32,35^ While all GelMA groups containing LPN had higher viscosity and more shear-thinning behavior, concentrations of ≥2% (w/v) LPN demonstrated a high shear-thinning capacity suitable for extrusion bioprinting (n index ≤ 0.33) [Fig. 1(a)]. The increase in initial viscosity observed at low shear rates (0.1 s^−1^) with the addition of 1%–4% (w/v) LPN to 5%–15% GelMA is a result of LPN's self-organization via electrostatic interactions between individual nanoclay disks throughout the polymer networks; once a critical shear rate is reached, nanoclay and polymer chains realign enhancing shear-thinning capacity [Figs. 1(a-i)–1(a-iii)].^53,54^ A commercially available bioink from CELLINK (GelMA-C) and 10% GelMA with 2% LPN exhibited similar shear-thinning characteristics, with n-indices <0.25 (GelMA-C—0.06. GelMA-LPN—0.133), further illustrating GelMA-LPN's potential as a prospective bioink (Fig. S6). Furthermore, the shear-recovery (viscoelasticity) of GelMA was enhanced in the presence of 2% LPN compared to 5%–10% (w/v) GelMA alone which can be classified as a poorly shear-thinning and Newtonian like due to an *n* index closer to 1 (*n* index 0.72–0.80) [Fig. 1(a-iv)].

Previous studies of GelMA-LPN (10% GelMA + 2% LPN w/v) have shown enhanced mechanical properties with up to twofold increase in Young's modulus (35.3 ± 1.5 kPa) after 1.5 min cross-linking compared to GelMA controls.^29^ The current study demonstrated comparable mechanical properties of (37.5 ± 9.1 kPa) [Fig. 2(b-ii)] for the same composition and cross-linking time; however, the addition of 4% (w/v) LPN concentration resulted in decreased Young's modulus (15.0 ± 8.3 kPa) and cross-linking propagation [Figs. 2(b) and 2(c)]. This effect was even more pronounced at 15% GelMA, where the modulus of hydrogels decreased with LPN concentrations >1% (w/v) compared to lower concentrations of GelMA (5%–10% w/v). It is hypothesized that increases in LPN concentration partially inhibits cross-linking of 10%–15% (w/v) GelMA concentrations.^55^ It was evident in low (5% w/v) GelMA concentrations, the high LPN concentration increased Young's modulus and cross-linking propagation [Figs. 2(b-i) and 2(c-i)]. On the other hand, at high GelMA concentrations, the local concentration has already passed optimal, and the high LPN concentrations prevent the formation of a consistent crosslinked network [Figs. 2(b-ii)–2(b-iv) and 2(c-ii)–2(c-iv)]. Incorporating 2% LPN to GelMA 5%–15% (w/v) demonstrated Young's moduli ranging from ∼0.91 to 73 kPa [Figs. 2(b-i)–2(b-iii)]; these values cover a wide range of tissue stiffness, from physiological tissues such as breast and endothelial tissue (∼0.8–1.5 kPa) to stiffer tissues like cardiac or smooth muscle (∼20–50 kPa).^40–42^

Bioprinting GelMA without a rheological additive is possible; however, consistent shape fidelity is limited by printing conditions and GelMA concentration. All bioinks containing 2% LPN were found to be printable and maintained their shape at room temperature (25 °C) except for 5% GelMA. It is expected that if the bioprinting temperature were raised to 37 °C, the print fidelity would likely diminish due to the temperature-sensitive nature of GelMA. One strategy to improve shape fidelity to achieve sol-gel transition is to regulate printing temperatures, with temperatures ranging from 21 to 27.5 °C, and a build plate temperature of ≤10 °C have been shown to thermally gelate the deposited GelMA fibers following extrusion.^56,57^ However, the use of straight nozzles during printing has been associated with increased shear stress on cells compared to conical nozzles.^11,16,58^ In the past decade, bioprinting technology has advanced, and many commercially available printers are now capable of thermoregulating both syringe and build plates, as well as cross-linking during ink extrusion, between layers, or after printing. Addition of 2% (w/v) LPN to GelMA significantly reduced temperature-dependent fluctuations of bioink viscosity [Fig. 2(c)], suggesting improved and more consistent printability.^3^ The electrostatic interaction of GelMA and LPN has been shown to influence thermal stability with increased zeta potential reported (−13 ± 2.63 to −34.4 ± 0.9 mV) with 0.5%–2% (w/v) LPN concentration compared to 5% GelMA control (−11.3 ± 0.4 mV).^42^ The negative charge interaction promotes LPN particle dispersion within GelMA polymer solutions and reduced temperature fluctuations [Figs. 2(d-i)–2(d-iv)]. This is important as the bioink temperature may change during the printing process, potentially affecting print fidelity and reproducibility. Based on the rheological evaluation and preliminary printing tests, 10% GelMA with 2% (w/v) LPN was determined to possess the ideal rheological properties for extrusion-based bioprinting and offers a highly tunable range of mechanical stiffness via visible light cross-linking.

In this study, the nozzle size used for bioprinting (ranging from 22 to 27 G) had limited effect on the behavior of MCF-7 cells in the printed constructs. The metabolic activity increased in 25–27 G groups over 7 days compared to 22 G nozzle size, while total number of cells and spheroid formation remained consistent in all groups [Figs. 4(a) and 4(b)]. Increasing the photocrosslinking duration of 1 min resulted in an increased stiffness (11.67 kPa) and 14 mW/cm^2^ light intensity limiting spheroid size and aggregate cell clusters (Fig. S1). The biocompatibility was further investigated and compared to 10% (w/v) GelMA droplets with matched stiffness values (1 kPa, Fig. S1). The study determined the bioprinting and addition of 2% (w/v) LPN enabled the generation of metabolically active spheroids and cell aggregates while 10% (w/v) GelMA control produced slightly larger spheroids (1315 ± 216 *μ*m) compared GelMA-LPN (1183 ± 167 *μ*m).

While GelMA-LPN demonstrates its suitability as a bioink, LPN does introduce some challenges. During fluorescence imaging of constructs, we observed a high level of unspecific background fluorescence throughout hydrogel construct stained with 4′,6-diamidino-2-phenylindole (DAPI), PI or Alexa Fluor™ 633 phalloidin in 405 and 633 nm channels (Fig. S3). The selection of buffer to disperse LPN particles as a result of increased levels of ions including Na^+^, Ca^2+^ and Mg^+^ reduces particle dispersion previously characterized using x-ray diffraction (XRD), energy-dispersive x-ray (EDX) and thixotropic behavior.^44,50^ A variety of mixing methods and buffers were tested to reduce background fluorescence and improve homogeneity of LPN incorporation to GelMA (Fig. S4). Dissolution of lyophilized GelMA and LPN powder in 100 mM HEPES at 37 °C using a magnetic stirrer overnight resulted in consistent fluorescence intensity in 488 nm channels throughout Teflon-cast and printed hydrogels using fluorescein isothiocyanate (FITC)-GelMA, suggesting good mixing (Fig. S4). The unspecific background fluorescence in GelMA-LPN constructs was persistent and more pronounced when the Laponite (LPN) particles were homogeneously dispersed throughout GelMA, rather than being clustered (Figs. S3 and S5). The formation of LPN within different aqueous fluids [de-ionized water, PBS, and fetal bovine serum (FBS)] influences its “house of cards” structure altering particle dispersion and hierarchical structure, as well as increased electrostatic interaction due to negatively charged surfaces and pH effects.^59,60^ Previous studies have investigated LPN's fluorescent and absorption interaction with organic dyes such as crystal violet and Nile Red and found LPN absorption-induced reactions and spectral shifts of intensity at 630 nm.^42,54,61,62^ Previous studies have established that LPN electrostatically interacts with GelMA's polymer chains, influenced by the positive amino acids and slightly negative carboxylic acid groups, which promotes enhanced particle distribution throughout polymer network.^52,63^ Similar background fluorescence was observed in 10% (v/v) PEGDA with the addition of 2% (w/v) LPN encapsulated with and without MCF-7 cells (Fig. S5). The cause of the background fluorescence remains to be determined. While this poses an issue for traditional live/dead staining, a combination of live-cell stain (FDA or Calcein AM) and brightfield images, or live cell tracking dyes have been previously shown effective alternatives and polymerase chain reaction, RNA sequencing type assays have been minimally affected.^25,35,37,60^ While GelMA-LPN has demonstrated high cytocompatibility in our study with MCF-7 cells, this is a resilient cancer cell line which does not imply that it will be cytocompatible with all cell types. Other studies have shown cytocompatibility with pre-osteoblasts (NIH MC3T3), fibroblasts (NIH-3T3), epithelial (MIA PaCa-2) and human bone marrow stromal cells (hBMSC's), but further cytocompatibility studies using sensitive patient-derived cells are needed to expand GelMA-LPN's versatility as a bioink for extrusion-based bioprinting.^30,35,64^

The incorporation of a rheological factorial approach offers insight into light-activated bioink properties utilizing four main testing profiles: viscosity shear rate profile, 3-interval viscosity thixotropy, rotational oscillatory tests with visible light cross-linking and temperature profile, as an effective prescreening and concentration optimizer. It was revealed that the addition of LPN enhanced GelMA's critical rheological properties required for extrusion based bioprinting with LPN 2% (w/v) being the optimal additive concentration. The inclusion of LPN to GelMA increased its printability and allowed for culture of metabolically active spheroids. This study illustrated an effective rheological approach for the characterization of GelMA-LPN bioinks which enhanced GelMA's biofabrication window while maintaining high cellular viability. The current study has also narrowed the LPN concentration range previously utilized with gelatin-based bioinks to 1%–2% (w/v) LPN for extrusion-based bioprinting, based on cross-linking inhibition and negative effects on mechanical properties at 4% (w/v) LPN. Future research should further explore GelMA-LPN's physiochemical interactions and LPN concentration range for tissue-specific applications.

## IV. METHODS

### A. Bioink development

Gelatin Methacryloyl (GelMA; porcine skin, Type A, 80% degree of functionalisation) was obtained by Gelomics Pty Ltd (Brisbane, Australia) and Laponite^®^ RD (LPN) was obtained from BYK (Wesel, Germany). Laponite powder, glass vials (Lab Supply, Australia) and Teflon stir bars (Thermofisher, Waltham, MA, United States) were first autoclaved (121 °C for 20 min). Lyophilized fluorescein isothiocyanate (FITC) labeled-GelMA and unlabeled GelMA (5%, 10%, or 15% w/v) containing 0%, 1%, 2%, or 4% LPN, respectively, was aseptically added and dissolved in phosphate-buffered saline solution (PBS) or 100 mM HEPES (Life Technologies, Thermofisher, Waltham, MA, United States) containing 0.15% lithium phenyl-2,4,6-trimethylbenzoylphosphinate (LAP) photoinitiator on a magnetic stirrer set to 300 rpm overnight at 37 °C. Poly(ethylene glycol) diacrylate (PEGDA, 700 Mn) (Sigma Aldrich) was prepared at a final concentration of 10% (v/v) in 100 mM HEPES containing 0.15% (w/v) LAP photoinitiator.

### B. Rheology

Rheological measurements were performed using a MCR302 rheometer (Anton Paar, Graz, Austria) through a series of rotational and oscillatory tests with cone-plate (CP) (25 mm diameter, 2° cone angle, 60 *μ*m truncation height, and 0.05 mm fixed gap) and parallel-plate (PP) (25 mm diameter and 0.15 mm fixed gap). All testing was performed at 25 °C unless otherwise stated. A CP geometry was utilized for viscosity profiles using a logarithmic shear rate ramp to determine shear-thinning properties. To determine the materials linear viscoelastic range (LVER) logarithmic ramps of strain and frequency (0.1%–1000% or 0.1 Hz) were performed [Fig. S1(a)]. 3-interval thixotropy test (3TT) viscosity and rotational oscillatory tests (photorheology and temperature profile) were performed within each sample's LVER range. Photorheology was performed using a LED light (405 nm) source placed at a set distance (6 cm for 32 mW/cm^2^ and 14 cm for 14 mW/cm^2^) from the rheometer plate [Figs. S1(b) and S1(c)]. A light intensity meter (Volumetric, BICO, Gothenburg, Sweden) was used to measure intensity prior to loading precursor solution. The shear-thinning properties were further evaluated using a power law equation (1) to define flow behavior, and Young's modulus (2) was calculated for photorheology experiments,

$$\sigma =K\;\dot{\gamma^{n}},$$ (1)

where K (Pa s) is the consistency index and n is the power law index,

$$E={2G}^{*}(1+v),$$ (2)

where ${2G}^{*}$ (Pa) is the complex shear modulus and v is the Poisson's ratio (GelMA = 0.44).^7,46^

### C. Cell culture

The MCF-7 human breast cancer cell line (CellBank, Sydney, Australia) was cultured in RPMI supplemented with 10% FBS, 1% (v/v) penicillin–streptomycin (Gibco, Thermofisher), 1% (v/v) MEM Non-Essential Amino Acids Solution (Gibco, Thermofisher), 1% (v/v) Sodium Pyruvate (Gibco, Thermofisher) and 0.1% (v/v) Insulin transferrin-selenium (Gibco, Thermofisher). MCF-7 cells were seeded in T75 flasks (Nunc, Thermofisher) and cultured to 70%–80% confluency in a cell culture incubator (37 °C, 5% CO_2_) and monitored daily using brightfield microscopy (Nikon) to assess morphology, with media changes performed every 2–3 days.

### D. 3D cell culture

In all experiments, MCF-7 cells were used between passages 19–20. Cells were washed with PBS prior to detachment with 0.25% (v/v) trypsin EDTA (Gibco, Thermofisher) and counted using an automated cell counter (Invitrogen, Thermofisher) with 0.4% (v/v) trypan blue solution (Gibco, Thermofisher). MCF-7 cells were encapsulated in 5%–10% (w/v) GelMA with or without 2% (w/v) LPN to produce 20 *μ*l free-swelling droplets with a cell density of 1 × 10^6^ cells/ml and cultured in 48-well plates (Thermofisher). All hydrogels were crosslinked for 30 s or 1 min to match stiffness using the LunaCrosslinker™ Visible Light Crosslinking System (Gelomics Pty Ltd), submerged in cell culture media and incubated at 37 °C with 5% CO_2_.

### E. 3D printing

A BIO X (CELLINK, Gothenburg, Sweden) 3D Bioprinter was used for all printing of bioink formulations at room temperature (RT) (25 °C). GelMA-LPN formulations were prepared the day prior and heated to 37 °C for 1 h before loading into a 3 ml syringe (Nordson, Westlake, Ohio, United States). All construct designs were developed using Inventor Professional (Autodesk) or custom G-codes. Files were then exported as .stl files and sliced using BioCAD (BIO X software, CELLINK). Printability index of selected bioinks was assessed using 15 × 15 × 3 mm^3^ lattice structures with various infill densities (6–15%) and printed using smooth flow tapered nozzles 22 (400 *μ*m), 25 (250 *μ*m), and 27 G (200 *μ*m) (Nordson). Once printed, each construct was crosslinked using the LunaCrosslinker (Gelomics Pty Ltd) for 1 min and then imaged using a stereomicroscope (Nikon, Tokyo, Japan); images were analyzed using Image J (National Institutes of Health, Bethesda, MA, USA) to quantify fiber diameter, pore size, and printability (Pr)

$$Pr=\frac{L^{2}}{16A},$$ (3)

where L is internal perimeter and A is internal area of each pore.^18^

For cell studies, a mixing adaptor^13^ was 3D-printed using an Asiga PRO 4K digital lithography printer (Alexandria, Australia) with DentaGuide resin (Asiga) and sterilized with 70% w/v ethanol for 30 min and dried prior to achieving a homogenous mixture of bioink and cells. GelMA (10.8%) and (LPN 2.5%) were heated to 37 °C and loaded into a 3 ml syringe (Terumo™, Thermofisher). MCF-7 cells were prepared at the same cell density as 3D hydrogel models (3 × 10^6^ cells) in 200 *μ*l of culture media and loaded into 3 ml syringe at 1:15 dilution to achieve final bioink concentration of 10% GelMA with 2% (w/v) LPN and final cell concentration of 1 × 10^6^, using a 3D-printed mixing unit to achieve homogeneity and loaded into 3 ml cartridge. The bioinks were printed with tapered conical nozzles (22, 25, and 27 G) at room temperature (25 °C), extrusion speeds of 12–20 mm/s, a pressure of 70–85 kPa and 15% infill density. Scaffolds of 10 × 10 mm^2^ size and 3–5 layers height were crosslinked post-printing using the LunaCrosslinker™ Visible Light Crosslinking System for 30 s.

### F. Cell viability

The viability of cell-laden constructs was assessed using Fluorescein Diacetate only (FDA, final concentration of 10 *μ*g/ml) (Thermofisher) in PBS. Unspecific background fluorescence was observed using Propidium Iodide (PI, final concentration of 5 *μ*g/ml) (Thermofisher) and 4′,6-diamidino-2-phenylindole (DAPI, 1 *μ*g/ml, Thermofisher) (Figs. S3 and S5). Samples were imaged using Zeiss Axio Observer 7 (Jena, Germany) at 10× magnification for 3D volumetric images (z-stacks), with an average total depth of 150–200 and 1.5 *μ*m slices. Each sample had three regions of interest (ROI) imaged at random for quantification (Image J). Samples imaged with Axio Observer utilized Apotome function using x5 grid/slice function followed by deconvolution (Zen, Zeiss).

### G. Metabolic activity

The metabolic activity of MCF-7 in free-swelling and 3D-printed constructs was quantified using PrestoBlue Cell Viability Reagent (Invitrogen, Thermofisher) following the manufacturer's recommendation. The reagent solution was prepared at a 1:10 ratio (PrestoBlue: cell culture media), 500 *μ*l of reagent solution was aliquoted per well and incubated for 45 min. Triplicates of 100 *μ*l from each sample were aliquoted into a 96-well plate, and absorbance was read at 590 nm using a microplate reader (BMG LABTECH, Ortenberg, Germany) using two different gains (500 and 900).

### H. Fluorescence staining

MCF-7 containing samples were fixed with 4% paraformaldehyde (Sigma Aldrich) at day 1 and 7, respectively, for 45 min at room temperature followed by blocking and permeabilisation using 5% (v/v) goat serum (Gibco, Thermofisher) and 0.01% (v/v) Triton X-100 (Sigma Aldrich) in PBS for a minimum of 2 h. First, samples were stained with Alexa Fluor 488 or 633 phalloidin (1:400 dilution; Invitrogen, Thermofisher) overnight at 4 °C on a shaker. Next, samples were washed three times in washing buffer (PBS with 1% v/v goat serum and 0.01% v/v Triton X-100) over 8 h at 4 °C on a shaker. Samples were then incubated with 4′,6-diamidino-2-phenylindole (DAPI, 1 *μ*g/ml, Thermofisher) and CellMask™ Orange (5 *μ*g/ml, Invitrogen, Thermofisher) overnight at 4 °C on a shaker. The following day, samples were washed for a further 8 h with washing buffer and stored in PBS at 4 °C until imaging.

### I. Statistical and data analysis

Statistical analysis was performed using GraphPad Prism v9.2. Statistical significance was determined using one-way or two-way analysis of variance (ANOVA), as appropriate, and followed by post-hoc tests (Tukey or Bonferroni multiple comparison tests). A p-value of less than 0.05 was considered statistically significant with asterisks denoting statistical value (^*^P < 0.05; ^**^P < 0.01; ^***^P < 0.001). Interaction terms were listed in appropriate figures unless stated otherwise. All experiments were performed with a sample size of n ≥ 3 and described within each figure caption. Data are presented using bar graphs with mean ± standard deviation (SD).

## SUPPLEMENTARY MATERIAL

See the supplementary material for the following details: amplitude oscillatory shear and photorheology of 5%–15% GelMA with 0%–4% (w/v) LPN at different light intensities (14 and 32 mW/cm^2^) (Fig. S1); 3D cell culture cytocompatibility of MCF-7 breast cancer cell line encapsulated in 5% GelMA and 0%–2% (w/v) LPN over 7 days (Fig. S2); fluorescence of 0%–2% LPN with 5%–15% (w/v) GelMA cell-laden (MCF-7 breast cancer cell line) hydrogels during different cell experiments at varied timepoints live and fixed samples (Fig. S3); the optimization of mixing methods and homogeneity of 5%–15% fluorescein isothiocyanate (FITC) labeled GelMA and 2% (w/v) LPN formulations in varied buffer solutions experiments at varied timepoints live and fixed samples (Fig. S4); background fluorescence of 0%–2% LPN with 10% (w/v) GelMA and 10% (v/v) PEGDA with cell-laden (MCF-7 breast cancer cell line) hydrogels at day 0 timepoint (Fig. S5); viscosity as a function of shear rate (0.1–1000 s^−1^) comparison of commercial bioink GelMA-C (CELLINK) and 10% GelMA 2% LPN (Fig. S6).

## Data Availability

The data that support the findings of this study are available from the corresponding authors upon reasonable request.

## References

[1] J. Groll , T. Boland , T. Blunk , J. A. Burdick , D.-W. Cho , P. D. Dalton *et al.*, “ Biofabrication: Reappraising the definition of an evolving field,” Biofabrication 8(1), 013001 (2016). 10.1088/1758-5090/8/1/013001 26744832

[2] J. Malda , J. Visser , F. P. Melchels , T. Jüngst , W. E. Hennink , W. J. Dhert *et al.*, “ 25th anniversary article: Engineering hydrogels for biofabrication,” Adv. Mater. 25(36), 5011–5028 (2013). 10.1002/adma.201302042 24038336

[3] R. Levato , T. Jungst , R. G. Scheuring , T. Blunk , J. Groll , and J. Malda , “ From shape to function: The next step in bioprinting,” Adv. Mater. 32(12), 1906423 (2020). 10.1002/adma.201906423 PMC7116209 32045053

[4] C. R. Alcala-Orozco , I. Mutreja , X. Cui , G. J. Hooper , K. S. Lim , and T. B. F. Woodfield , “ Hybrid biofabrication of 3D osteoconductive constructs comprising Mg-based nanocomposites and cell-laden bioinks for bone repair,” Bone 154, 116198 (2022). 10.1016/j.bone.2021.116198 34534709

[5] L. Ouyang , J. P. K. Armstrong , Y. Lin , J. P. Wojciechowski , C. Lee-Reeves , D. Hachim *et al.*, “ Expanding and optimizing 3D bioprinting capabilities using complementary network bioinks,” Sci. Adv. 6(38), eabc5529 (2020). 10.1126/sciadv.abc5529 32948593 PMC7500929

[6] N. Majumder , A. Mishra , and S. Ghosh , “ Effect of varying cell densities on the rheological properties of the bioink,” Bioprinting 28, e00241 (2022). 10.1016/j.bprint.2022.e00241

[7] N. Monteiro , G. Thrivikraman , A. Athirasala , A. Tahayeri , C. M. França , J. L. Ferracane *et al.*, “ Photopolymerization of cell-laden gelatin methacryloyl hydrogels using a dental curing light for regenerative dentistry,” Dent. Mater. 34(3), 389–399 (2018). 10.1016/j.dental.2017.11.020 29199008 PMC5818302

[8] A. Herrera-Ruiz , B. B. Tovar , R. G. García , M. F. L. Tamez , and N. Mamidi , “ Nanomaterials-incorporated chemically modified gelatin methacryloyl-based biomedical composites: A novel approach for bone tissue engineering,” Pharmaceutics 14(12), 2645 (2022). 10.3390/pharmaceutics14122645 36559139 PMC9788194

[9] S. Heid and A. R. Boccaccini , “ Advancing bioinks for 3D bioprinting using reactive fillers: A review,” Acta Biomater. 113, 1–22 (2020). 10.1016/j.actbio.2020.06.040 32622053

[10] N. Rajabi , A. Rezaei , M. Kharaziha , H. R. Bakhsheshi-Rad , H. Luo , S. RamaKrishna *et al.*, “ Recent advances on bioprinted gelatin methacrylate-based hydrogels for tissue repair,” Tissue Eng., Part A 27(11–12), 679–702 (2021). 10.1089/ten.tea.2020.0350 33499750

[11] N. Paxton , W. Smolan , T. Böck , F. Melchels , J. Groll , and T. Jungst , “ Proposal to assess printability of bioinks for extrusion-based bioprinting and evaluation of rheological properties governing bioprintability,” Biofabrication 9(4), 044107 (2017). 10.1088/1758-5090/aa8dd8 28930091

[12] A. Ribeiro , M. M. Blokzijl , R. Levato , C. W. Visser , M. Castilho , W. E. Hennink *et al.*, “ Assessing bioink shape fidelity to aid material development in 3D bioprinting,” Biofabrication 10(1), 014102 (2018). 10.1088/1758-5090/aa90e2 PMC7116103 28976364

[13] S. Dani , T. Ahlfeld , F. Albrecht , S. Duin , P. Kluger , A. Lode *et al.*, “ Homogeneous and reproducible mixing of highly viscous biomaterial inks and cell suspensions to create bioinks,” Gels 7(4), 227 (2021). 10.3390/gels7040227 34842704 PMC8628813

[14] D. Chimene , R. Kaunas , and A. K. Gaharwar , “ Hydrogel bioink reinforcement for additive manufacturing: A focused review of emerging strategies,” Adv. Mater. 32(1), 1902026 (2020). 10.1002/adma.201902026 31599073

[15] L. Ouyang , “ Pushing the rheological and mechanical boundaries of extrusion-based 3D bioprinting,” Trends Biotechnol. 40(7), 891–902 (2022). 10.1016/j.tibtech.2022.01.001 35094846

[16] A. Blaeser , D. F. Duarte Campos , U. Puster , W. Richtering , M. M. Stevens , and H. Fischer , “ Controlling shear stress in 3D bioprinting is a key factor to balance printing resolution and stem cell integrity,” Adv. Healthcare Mater. 5(3), 326–333 (2016). 10.1002/adhm.201500677 26626828

[17] L. Ning , A. Guillemot , J. Zhao , G. Kipouros , and X. Chen , “ Influence of flow behavior of alginate–cell suspensions on cell viability and proliferation,” Tissue Eng., Part C 22(7), 652–662 (2016). 10.1089/ten.tec.2016.0011 27166436

[18] C. O'Connell , J. Ren , L. Pope , Y. Zhang , A. Mohandas , R. Blanchard *et al.*, “ Characterizing bioinks for extrusion bioprinting: Printability and rheology,” Methods Mol. Biol. 2140, 111–133 (2020). 10.1007/978-1-0716-0520-2_7 32207108

[19] F. P. W. Melchels , W. J. A. Dhert , D. W. Hutmacher , and J. Malda , “ Development and characterisation of a new bioink for additive tissue manufacturing,” J. Mater. Chem. B 2(16), 2282–2289 (2014). 10.1039/c3tb21280g 32261716

[20] T. Jungst , W. Smolan , K. Schacht , T. Scheibel , and J. Groll , “ Strategies and molecular design criteria for 3D printable hydrogels,” Chem. Rev. 116(3), 1496–1539 (2016). 10.1021/acs.chemrev.5b00303 26492834

[21] L. Ouyang , R. Yao , Y. Zhao , and W. Sun , “ Effect of bioink properties on printability and cell viability for 3D bioplotting of embryonic stem cells,” Biofabrication 8(3), 035020 (2016). 10.1088/1758-5090/8/3/035020 27634915

[22] A. Pössl , D. Hartzke , T. M. Schmidts , F. E. Runkel , and P. Schlupp , “ A targeted rheological bioink development guideline and its systematic correlation with printing behavior,” Biofabrication 13(3), 035021 (2021). 10.1088/1758-5090/abde1e 33472177

[23] L. Ouyang , C. B. Highley , W. Sun , and J. A. Burdick , “ A Generalizable strategy for the 3D bioprinting of hydrogels from nonviscous photo-crosslinkable inks,” Adv. Mater. 29(8), 1604983 (2017). 10.1002/adma.201604983 27982464

[24] C. B. Highley , C. B. Rodell , and J. A. Burdick , “ Direct 3D printing of shear-thinning hydrogels into self-healing hydrogels,” Adv. Mater. 27(34), 5075–5079 (2015). 10.1002/adma.201501234 26177925

[25] G. Cidonio , M. Glinka , Y. H. Kim , J. M. Kanczler , S. A. Lanham , T. Ahlfeld *et al.*, “ Nanoclay-based 3D printed scaffolds promote vascular ingrowth *ex vivo* and generate bone mineral tissue *in vitro* and *in vivo*,” Biofabrication 12(3), 035010 (2020). 10.1088/1758-5090/ab8753 32259804

[26] T. Gao , G. J. Gillispie , J. S. Copus , A. K. Pr , Y. J. Seol , A. Atala *et al.*, “ Optimization of gelatin-alginate composite bioink printability using rheological parameters: A systematic approach,” Biofabrication 10(3), 034106 (2018). 10.1088/1758-5090/aacdc7 29923501 PMC6040670

[27] M. I. Neves , L. Moroni , and C. C. Barrias , “ Modulating alginate hydrogels for improved biological performance as cellular 3D microenvironments,” Front. Bioeng. Biotechnol. 8, 665 (2020). 10.3389/fbioe.2020.00665 32695759 PMC7338591

[28] D. Chimene , L. Miller , L. M. Cross , M. K. Jaiswal , I. Singh , and A. K. Gaharwar , “ Nanoengineered osteoinductive bioink for 3D bioprinting bone tissue,” ACS Appl. Mater. Interfaces 12(14), 15976–15988 (2020). 10.1021/acsami.9b19037 32091189 PMC12833824

[29] D. Chimene , C. W. Peak , J. L. Gentry , J. K. Carrow , L. M. Cross , E. Mondragon *et al.*, “ Nanoengineered ionic–covalent entanglement (NICE) bioinks for 3D bioprinting,” ACS Appl. Mater. Interfaces 10(12), 9957–9968 (2018). 10.1021/acsami.7b19808 29461795

[30] A. A. Adib , A. Sheikhi , M. Shahhosseini , A. Simeunović , S. Wu , C. E. Castro *et al.*, “ Direct-write 3D printing and characterization of a GelMA-based biomaterial for intracorporeal tissue engineering,” Biofabrication 12(4), 045006 (2020). 10.1088/1758-5090/ab97a1 32464607

[31] V. H. Mouser , F. P. Melchels , J. Visser , W. J. Dhert , D. Gawlitta , and J. Malda , “ Yield stress determines bioprintability of hydrogels based on gelatin-methacryloyl and gellan gum for cartilage bioprinting,” Biofabrication 8(3), 035003 (2016). 10.1088/1758-5090/8/3/035003 27431733 PMC4954607

[32] L. Dong , Z. Bu , Y. Xiong , H. Zhang , J. Fang , H. Hu *et al.*, “ Facile extrusion 3D printing of gelatine methacrylate/Laponite nanocomposite hydrogel with high concentration nanoclay for bone tissue regeneration,” Int. J. Biol. Macromol. 188, 72–81 (2021). 10.1016/j.ijbiomac.2021.07.199 34364938

[33] M. A. Habib and B. Khoda , “ Development of clay based novel bio-ink for 3D bio-printing process,” Procedia Manuf. 26, 846–856 (2018). 10.1016/j.promfg.2018.07.105

[34] Y. J. Shin , R. T. Shafranek , J. H. Tsui , J. Walcott , A. Nelson , and D.-H. Kim , “ 3D bioprinting of mechanically tuned bioinks derived from cardiac decellularized extracellular matrix,” Acta Biomater. 119, 75–88 (2021). 10.1016/j.actbio.2020.11.006 33166713

[35] G. Cidonio , C. R. Alcala-Orozco , K. S. Lim , M. Glinka , I. Mutreja , Y. H. Kim *et al.*, “ Osteogenic and angiogenic tissue formation in high fidelity nanocomposite Laponite-gelatin bioinks,” Biofabrication 11(3), 035027 (2019). 10.1088/1758-5090/ab19fd 30991370

[36] J. I. Dawson , J. M. Kanczler , X. B. Yang , G. S. Attard , and R. O. C. Oreffo , “ Clay gels for the delivery of regenerative microenvironments,” Adv. Mater. 23(29), 3304–3308 (2011). 10.1002/adma.201100968 21661063

[37] J. I. Dawson and R. O. C. Oreffo , “ Clay: New opportunities for tissue regeneration and biomaterial design,” Adv. Mater. 25(30), 4069–4086 (2013). 10.1002/adma.201301034 23722321

[38] L. M. Cross , J. K. Carrow , X. Ding , K. A. Singh , and A. K. Gaharwar , “ Sustained and prolonged delivery of protein therapeutics from two-dimensional nanosilicates,” ACS Appl. Mater Interfaces 11(7), 6741–6750 (2019). 10.1021/acsami.8b17733 30676016 PMC6472961

[39] M. Ghadiri , H. Hau , W. Chrzanowski , H. Agus , and R. Rohanizadeh , “ Laponite clay as a carrier for *in situ* delivery of tetracycline,” RSC Adv. 3(43), 20193–20201 (2013). 10.1039/c3ra43217c

[40] M. Vigata , C. Meinert , S. Pahoff , N. Bock , and D. W. Hutmacher , “ Gelatin methacryloyl hydrogels control the localized delivery of albumin-bound paclitaxel,” Polymers 12(2), 501 (2020). 10.3390/polym12020501 32102478 PMC7077643

[41] Z. Ma , H. He , C. Deng , Y. Ren , D. Lu , W. Li *et al.*, “ 3D bioprinting of proangiogenic constructs with induced immunomodulatory microenvironments through a dual cross-linking procedure using Laponite incorporated bioink,” Composites, Part B 229, 109399 (2022). 10.1016/j.compositesb.2021.109399

[42] J. R. Xavier , T. Thakur , P. Desai , M. K. Jaiswal , N. Sears , E. Cosgriff-Hernandez *et al.*, “ Bioactive nanoengineered hydrogels for bone tissue engineering: A growth-factor-free approach,” ACS Nano 9(3), 3109–3118 (2015). 10.1021/nn507488s 25674809

[43] D. B. Andrade , L. L. S. Soares , F. L. A. Cardoso , I. S. Lima , J. G. V. Silva , M. A. M. Carvalho *et al.*, “ Hydrogel based on nanoclay and gelatin methacrylate polymeric matrix as a potential osteogenic application,” J. Funct. Biomater. 14(2), 74 (2023). 10.3390/jfb14020074 36826873 PMC9961749

[44] B. Liu , J. Li , X. Lei , S. Miao , S. Zhang , P. Cheng *et al.*, “ Cell-loaded injectable gelatin/alginate/LAPONITE® nanocomposite hydrogel promotes bone healing in a critical-size rat calvarial defect model,” RSC Adv. 10(43), 25652–25661 (2020). 10.1039/D0RA03040F 35518607 PMC9055310

[45] A. Paul , V. Manoharan , D. Krafft , A. Assmann , J. A. Uquillas , S. R. Shin *et al.*, “ Nanoengineered biomimetic hydrogels for guiding human stem cell osteogenesis in three dimensional microenvironments,” J. Mater. Chem. B 4(20), 3544–3554 (2016). 10.1039/C5TB02745D 27525102 PMC4980085

[46] S. R. Polio , A. N. Kundu , C. E. Dougan , N. P. Birch , D. E. Aurian-Blajeni , J. D. Schiffman *et al.*, “ Cross-platform mechanical characterization of lung tissue,” PLoS One 13(10), e0204765 (2018). 10.1371/journal.pone.0204765 30332434 PMC6192579

[47] M. Vigata , C. D. O'Connell , S. Cometta , D. W. Hutmacher , C. Meinert , and N. Bock , “ Gelatin methacryloyl hydrogels for the localized delivery of cefazolin,” Polymers 13(22), 3960 (2021). 10.3390/polym13223960 34833259 PMC8618379

[48] S. Kyle , Z. M. Jessop , A. Al-Sabah , and I. S. Whitaker , “ Printability' of candidate biomaterials for extrusion based 3D printing: State-of-the-art,” Adv. Healthcare Mater. 6(16), 1700264 (2017). 10.1002/adhm.201700264 28558161

[49] A. C. Daly , M. E. Prendergast , A. J. Hughes , and J. A. Burdick , “ Bioprinting for the biologist,” Cell 184(1), 18–32 (2021). 10.1016/j.cell.2020.12.002 33417859 PMC10335003

[50] A. Sheikhi , S. Afewerki , R. Oklu , A. K. Gaharwar , and A. Khademhosseini , “ Effect of ionic strength on shear-thinning nanoclay–polymer composite hydrogels,” Biomater. Sci. 6(8), 2073–2083 (2018). 10.1039/C8BM00469B 29944151 PMC6085890

[51] S. Afewerki , L. S. S. M. Magalhães , A. D. R. Silva , T. D. Stocco , E. C. Silva Filho , F. R. Marciano *et al.*, “ Bioprinting a synthetic smectic clay for orthopedic applications,” Adv. Healthcare Mater. 8(13), 1900158 (2019). 10.1002/adhm.201900158 30957992

[52] B. Ruzicka and E. Zaccarelli , “ A fresh look at the Laponite phase diagram,” Soft Matter 7(4), 1268–1286 (2011). 10.1039/c0sm00590h

[53] F.-F. Cai , S. Heid , and A. R. Boccaccini , “ Potential of Laponite® incorporated oxidized alginate–gelatin (ADA-GEL) composite hydrogels for extrusion-based 3D printing,” J. Biomed. Mater. Res., Part B 109(8), 1090–1104 (2021). 10.1002/jbm.b.34771 33277973

[54] H. Tomás , C. S. Alves , and J. Rodrigues , “ Laponite®: A key nanoplatform for biomedical applications?,” Nanomedicine: Nanotechnol., Biol. Med. 14(7), 2407–2420 (2018). 10.1016/j.nano.2017.04.016 28552649

[55] S. Morariu , M. Bercea , and C.-E. Brunchi , “ Influence of Laponite RD on the properties of poly(vinyl alcohol) hydrogels,” J. Appl. Polym. Sci. 135(35), 46661 (2018). 10.1002/app.46661

[56] T. Billiet , E. Gevaert , T. De Schryver , M. Cornelissen , and P. Dubruel , “ The 3D printing of gelatin methacrylamide cell-laden tissue-engineered constructs with high cell viability,” Biomaterials 35(1), 49–62 (2014). 10.1016/j.biomaterials.2013.09.078 24112804

[57] W. Liu , M. A. Heinrich , Y. Zhou , A. Akpek , N. Hu , X. Liu *et al.*, “ Extrusion bioprinting of shear-thinning gelatin methacryloyl bioinks,” Adv. Healthcare Mater. 6(12), e00093 (2017). 10.1002/adhm.201601451 PMC5545786 28464555

[58] S. Boularaoui , G. Al Hussein , K. A. Khan , N. Christoforou , and C. Stefanini , “ An overview of extrusion-based bioprinting with a focus on induced shear stress and its effect on cell viability,” Bioprinting 20, e00093 (2020). 10.1016/j.bprint.2020.e00093

[59] P. Shi , Y.-H. Kim , M. Mousa , R. R. Sanchez , R. O. C. Oreffo , and J. I. Dawson , “ Self-assembling nanoclay diffusion gels for bioactive osteogenic microenvironments,” Adv. Healthcare Mater. 7(15), 1800331 (2018). 10.1002/adhm.201800331 29911340

[60] M. Ghadiri , W. Chrzanowski , W. H. Lee , A. Fathi , F. Dehghani , and R. Rohanizadeh , “ Physico-chemical, mechanical and cytotoxicity characterizations of Laponite^®^/alginate nanocomposite,” Appl. Clay Sci. 85, 64–73 (2013). 10.1016/j.clay.2013.08.049

[61] M. Grabolle , M. Starke , and U. Resch-Genger , “ Highly fluorescent dye–nanoclay hybrid materials made from different dye classes,” Langmuir 32(14), 3506–3513 (2016). 10.1021/acs.langmuir.5b04297 27007448

[62] C. Ley , J. Brendlé , A. Walter , P. Jacques , A. Ibrahim , and X. Allonas , “ On the interaction of triarylmethane dye crystal violet with LAPONITE^®^ clay: Using mineral nanoparticles to control the dye photophysics,” Phys. Chem. Chem. Phys. 17(26), 16677–16681 (2015). 10.1039/C5CP02370J 26028222

[63] M. A. Sakr , K. Sakthivel , T. Hossain , S. R. Shin , S. Siddiqua , J. Kim *et al.*, “ Recent trends in gelatin methacryloyl nanocomposite hydrogels for tissue engineering,” J. Biomed. Mater. Res., Part A 110(3), 708–724 (2022). 10.1002/jbm.a.37310 34558808

[64] N. R. de Barros , A. Gomez , M. Ermis , N. Falcone , R. Haghniaz , P. Young *et al.*, “ Gelatin methacryloyl and Laponite bioink for 3D bioprinted organotypic tumor modeling,” Biofabrication 15(4), 045005 (2023). 10.1088/1758-5090/ace0db PMC10683563 37348491

